# Regulation of Krüppel-Like Factor 15 Expression by Herpes Simplex Virus Type 1 or Bovine Herpesvirus 1 Productive Infection

**DOI:** 10.3390/v13061148

**Published:** 2021-06-15

**Authors:** Fouad S. El-mayet, Kelly S. Harrison, Clinton Jones

**Affiliations:** 1 Department of Veterinary Pathobiology, College of Veterinary Medicine, Oklahoma State University, Stillwater, OK 74078, USA; fouad.saad@fvtm.bu.edu.eg (F.S.E.-m.); hkellys@okstate.edu (K.S.H.); 2 Department of Virology, Faculty of Veterinary Medicine, Benha University, Moshtohor 13736, Kaliobyia, Egypt

**Keywords:** herpes simplex virus type 1 (HSV-1), bovine herpesvirus 1 (BoHV-1), Krüppel-like factor 15 (KLF15), infected cell protein 0 (ICP0), BoHV-1 ICP0 (bICP0)

## Abstract

Expression of Krüppel-like factor 15 (KLF15), a stress-induced transcription factor, is induced during bovine herpesvirus 1 (BoHV-1) reactivation from latency, and KLF15 stimulates BoHV-1 replication. Transient transfection studies revealed that KLF15 and glucocorticoid receptor (GR) cooperatively transactivate the BoHV-1-immediate-early transcription unit 1 (IEtu1), herpes simplex virus type 1 (HSV-1) infected cell protein 0 (ICP0), and ICP4 promoters. The IEtu1 promoter drives expression of bICP0 and bICP4, two key BoHV-1 transcriptional regulatory proteins. Based on these studies, we hypothesized infection is a stressful stimulus that increases KLF15 expression and enhances productive infection. New studies demonstrated that silencing KLF15 impaired HSV-1 productive infection, and KLF15 steady-state protein levels were increased at late stages of productive infection. KLF15 was primarily localized to the nucleus following infection of cultured cells with HSV-1, but not BoHV-1. When cells were transfected with a KLF15 promoter construct and then infected with HSV-1, promoter activity was significantly increased. The ICP0 gene, and to a lesser extent, bICP0 transactivated the KLF15 promoter in the absence of other viral proteins. In contrast, BoHV-1 or HSV-1 encoded VP16 had no effect on KLF15 promoter activity. Collectively, these studies revealed that HSV-1 and BoHV-1 productive infection increased KLF15 steady-state protein levels, which correlated with increased virus production.

## 1. Introduction

Bovine herpesvirus 1 (BoHV-1) and herpes simplex virus type 1 (HSV-1) are important pathogens of their respective hosts [1]. Productive infection leads to high levels of virus production and viral transmission. Viral genes are expressed in three distinct phases: immediate-early (IE), early, and late [2,3]. The tegument protein (VP16) specifically activates IE promoters by interacting with two cellular proteins, Oct-1 and HCF-1 [4,5,6]. Infected cell protein 0 (ICP0) and ICP4 are key viral transcriptional regulatory proteins that drive early and late gene expression. Three BoHV-1-immediate-early (IE) genes express mRNA translated into infected cell protein 0 (bICP0), bICP4, and bICP22 [7,8]. While the expression of HSV-1-encoded ICP0 and ICP4 is regulated by separate promoters, the BoHV-1-immediate-early transcription unit 1 (IEtu1) promoter controls the IE expression of a single mRNA that is alternatively spliced to produce bICP0 and bICP4 proteins [7,8,9]. A separate bICP0 E promoter sustains bICP0 expression throughout productive infection [9]. During productive infection, BoHV-1 and HSV-1 encode early and late genes that generally have similar functions.

Following acute infection of mucosal surfaces, BoHV-1 and HSV-1 establish a life-long latent infection in neurons [10,11,12]. In contrast to productive infection, viral gene expression in latently infected neurons is restricted to the BoHV-1-encoded latency-related (LR) gene or HSV-1-latency-associated transcript (LAT). These genes encode multiple products that promote neuronal survival by inhibiting apoptosis and the expression of viral regulatory genes important for productive infection [13,14,15,16]. The LR gene encodes a protein (ORF2) that inhibits apoptosis [13], whereas LAT appears to be a non-protein-coding locus, reviewed in [17]. LR gene products and LAT are predicted to promote reactivation from latency by maintaining a pool of latently infected neurons that can successfully reactivate from latency. Stress, in general, increases the frequency of BoHV-1 [11,18] and HSV-1 [19,20] reactivation from latency. The synthetic corticosteroid dexamethasone (DEX) triggers BoHV-1 reactivation from latency, reviewed in [21], and accelerates HSV-1 explant induced reactivation [22,23]. BoHV-1 [24,25] and HSV-1 [26,27] productive infection is also stimulated by DEX. Corticosteroids bind and activate the glucocorticoid receptor (GR) and mineralocorticoid receptor (MR) [28], suggesting these nuclear hormone receptors directly stimulate reactivation from latency.

Cellular transcription factors were identified in TG neurons within the first three hours following DEX treatment of BoHV-1 latently infected calves [29]. Several members of the Krüppel-like factor (KLF) family of transcription factors [30] were identified in this study, including KLF15 [29]. Additionally, explants of mouse TG contain more TG neurons that express KLF15 if the tissue is incubated with DEX [31]. Furthermore, the HSV-1 ICP0 promoter is cooperatively transactivated by GR and KLF15, and productive infection is impaired when cells are treated with a GR-specific antagonist [27]. Since GR and KLF15 stimulate BoHV-1 productive infection [25] and transactivates key viral promoters, stressful stimuli can trigger reactivation from latency.

The primary objectives of this study were to test what effects BoHV-1 and HSV-1 had on KLF15 expression and whether KLF15 mediates productive infection. Additional studies examined the effect viral infection has on KLF15 subcellular localization and whether viral genes transactivated the KLF15 promoter activity.

## 2. Materials and Methods

### 2.1. Cells and Viruses

Murine neuroblastoma (Neuro-2A), human neuroblastoma (SH-SY5Y), and Vero cells were grown in Minimal Essential Media (MEM) supplemented with 5% fetal bovine serum (FBS). Madin-Darby bovine kidney cells (MDBK) were grown in MEM supplemented with 10% FBS. All media contained penicillin (10 U/mL) and streptomycin (100 µg/mL).

The BoHV-1 Cooper strain (wt virus) is the North American prototype strain and was obtained from the National Veterinary Services Laboratory, Animal and Plant Health Inspection Services, Ames, Iowa. Stock cultures of BoHV-1 were prepared in CRIB or MDBK cells. The HSV-1 McKrae strain was obtained from the late Dr. Steven Wechsler (University of California, Irvine, CA, USA), and stock cultures were prepared in Vero cells.

### 2.2. Plasmids

A 4.3 kb fragment of the human KLF15 promoter upstream of the ATG start codon was subcloned into the KpnI/EcoRV cloning sites of the pGL4.20 firefly luciferase reporter plasmid (Promega, Madison, WI, USA) to generate pGL4.20-hKLF15 [32]. The bICP0 expression construct was obtained from M. Schwytzer (Zürich, Switzerland). The HSV-1 ICP0 expression construct was obtained from P. Schaffer (Harvard University). The VP16 open reading frames for BoHV-1 and HSV-1 were synthesized by GenScript and cloned into a Flag-tagged vector such that these constructs express a Flag-tagged VP16 protein.

### 2.3. SDS-Polyacrylamide Gels and Western Blots

The designated cultured cells were infected with wt HSV-1 or BoHV-1 at a multiplicity of infection (MOI) of 1 PFU/cell and cell lysate, collected at various times (hours) after infection. Cultures were washed with PBS and suspended in RIPA lysis buffer (50 mM Tris HCL (pH 8.0), 150 mM NaCl, 2 mM EDTA (pH 8.0), 1% NP-40, 0.5% sodium deoxycholate, 0.1% SDS), and protease inhibitor (Pierce Biotechnology, Rockford, lL, USA). The cell lysate was incubated on ice for 30 min, sonicated, and then clarified by centrifugation at 15,000× *g* at 4 °C for 15 min. Protein concentrations were quantified by the Bradford assay (Bio-Rad, Hercules, CA, USA). For SDS-PAGE, proteins were mixed with an equal amount of 2× sample-loading buffer (62.5 mM Tris-HCl [pH 6.8], 2% SDS, 50 mM dithiothreitol, 0.1% bromophenol blue, 10% glycerol) and boiled for 5 min. Proteins were separated in 10% SDS–PAGE gels. After electrophoresis, proteins were transferred onto a polyvinylidene difluoride membrane (Immobilon-P; Millipore, Burlington, MA, USA) and blocked for 1 h in 5% *w*/*v* nonfat dry milk with 1× Tris-buffered saline—0.1% Tween 20 (TBS-T). Membranes were incubated with the designated primary antibody at 4 °C with gentle shaking overnight. The primary antibody was diluted 1:1000 in blocking solution. Antibodies directed against b-tubulin (Fisher Scientific, Waltham, MA, USA; catalog no. MA5-16308) and Glyceraldehyde-3-Phosphate Dehydrogenase (GAPDH; Santa Cruz Biotechnology, Santa Cruz, CA, USA) was used as a loading control. The KLF15 primary antibody (ab167192) and ICP4 antibody were purchased from (Abcam, Cambridge, MA, USA; ab6514). A peptide-specific rabbit antibody directed against bICP4 was produced in rabbits by Affinity Bioreagents (Golden, CO, USA). The secondary donkey anti-rabbit antibody (NA9340V) was purchased from (GE Healthcare, Piscataway, NJ, USA) and the secondary sheep anti-mouse antibody was purchased from GE Healthcare. After 45 min of washing with TBS-T, blots were incubated with secondary antibodies (peroxidase-conjugated immunoglobulin G (Amersham Biosciences, Piscataway NJ, USA), which was diluted 1:2000 in 5% nonfat milk in TBS-T for 1 h. Blots were washed 45 min with TBS-T, exposed to Amersham ECL reagents, and imaged using an Amersham imager 600 (GE).

### 2.4. Nuclear and Cytoplasmic Fractionation

Vero or MDBK cells were infected with wt HSV-1 or BoHV-1 at an MOI of 1 PFU/cell for 8, 16, or 24 h after infection. The ReadyPrep™ Protein Extraction Kit (Cytoplasmic/Nuclear) was purchased from Bio-Rad, USA (catalog no. 163–2089) and used to separate cytoplasmic proteins from nuclear proteins. Cells were harvested by centrifugation at 2500× *g* for 5 min and washed twice in cold phosphate-buffered saline (PBS). Cells were suspended in ice-cold cytoplasmic protein extraction buffer (CPEB) with protease inhibitors and incubated on ice for 30 min. Cell suspensions were gently passed through a 20-gauge needle (10–20 strokes) to lyse cells without damaging the nuclei. Cell lysate was centrifuged at 1000× *g* for 10 min at 4 °C. The supernatant containing cytoplasmic proteins was immediately transferred to a new tube (on ice). The remaining nuclear pellet was washed one time with CPEB, and the supernatant was discarded. The nuclear pellet was suspended in freshly prepared protein solubilization buffer (PSB) and vortexed 4–5 times, 60 s each, to solubilize the nuclear proteins. Centrifugation (16,000× *g*) was performed for 15–20 min at room temperature to pellet genomic DNA and other debris. The clarified supernatant was transferred into a new microcentrifuge tube labeled Nuclear Protein Fraction. Protein concentrations were quantified by the Bradford assay, and standard 10% SDS-polyacrylamide gels were used to analyze KLF15 protein levels. An antibody directed against histone H3 (Abcam; ab1191) was diluted at 1:500 and used as a control for fractionation studies.

### 2.5. Immunofluorescence

Vero or MDBK cells seeded into 2-well chamber slides (Nunc. Inc., Naperville, IL, USA) were incubated in MEM supplemented with 10% FBS at 37 °C, 5% CO_2_ for 24 h. Cells were mock-infected or infected with HSV-1 or BoHV-1 at an MOI of 1 for 16 h. Cells were fixed in 4% paraformaldehyde in PBS pH 7.4 for 10 min at room temperature, and permeabilized with 0.25% Triton × −100 in PBS pH 7.4 for 10 min at room temperature, blocked with 1% BSA in PBST (PBS + 0.1% Tween 20) for 30 min, and incubated with anti-KLF15 antibody (Abcam, ab167192, at a concentration of 10 µg/mL) in 1% BSA in PBST for 12 h. After three washes, cells were incubated with Alexa Fluor 488 goat anti-mouse IgG (H+L, Invitrogen, Waltham, MA, USA; A-11001, 1:500 dilution) for 1 h in the dark. After three washes, DAPI (4′,6-diamidino-2-phenylindole) staining was performed to visualize the nucleus. Slides were covered with coverslips by using a Gel Mount aqueous mounting medium (Electron Microscopy Sciences by Fisher Scientific, Waltham, MA, USA). Images were obtained by confocal microscopy (Leica, Wetzlar, Germany).

### 2.6. Transfection and Dual-Luciferase Reporter Assays

For productive infection assays, Vero cells were transfected with a reporter construct containing the human KLF15 promoter (pGL4.20-hKLF15) obtained kindly from Dr. Yiqing Guo (Stony Brook University, New York, NY, USA). Lipofectamine 3000 (catalog no. L3000075; Invitrogen) was used according to the manufacturer’s instructions. An empty vector was added as needed. Twenty-four hours later, cells were infected with BoHV-1 or HSV-1 at an MOI of 0.1 or 0.5 for 24 h. Cells were harvested and protein lysate subjected to a dual-luciferase assay using a commercially available kit (E1910; Promega, Madison, WI, USA). Luminescence was measured using a GloMax 20/20 luminometer (E5331; Promega). Data for the luciferase activity were averaged from the results of multiple transfections experiments (at least three independent experiments were performed).

### 2.7. Analysis of KLF15 and Scrambled siRNA on Cell Viability and Productive Infection

A mouse-specific KLF15 siRNA (SR413764, Locus ID 66277) was purchased from OriGene Technologies (Rockville, MD, USA). A universal scrambled negative control siRNA was included (SR30004). Both siRNAs were reconstituted in an RNAse-free suspension buffer. Neuro-2A cells were grown in MEM containing 2% charcoal-stripped FBS and transfected with increasing concentrations of siRNA duplexes using Lipofectamine 3000 according to manufacturer instructions. Transfections were incubated at 37 °C, 5% CO_2_ for 48 h prior to trypan blue exclusion assay using the BioRad TC20 automated cell counter.

Neuro-2A cells were grown as above and transfected with increasing concentrations of KLF15 siRNA duplexes using Lipofectamine 3000. The universal scrambled negative control siRNA (25 µm) was included as a control. Cells were then infected with HSV-1 at an MOI of 1 for 1 h at 37 °C, 5% CO_2_ with rocking. Media was replaced, and infections incubated for 24 h. Media and cells were harvested, freeze-thawed at −80/37 °C three times prior to total virus enumeration using plaque assay, as described previously [27,33].

## 3. Results

### 3.1. Silencing KLF15 Significantly Reduced HSV-1 Productive Infection

**Figure 1:**
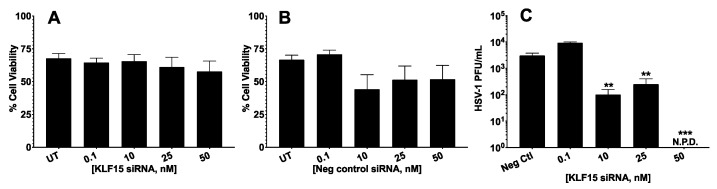
KLF15 siRNA reduces HSV-1 infection in Neuro-2A cells. KLF15 (Panel **A**) or the universal scrambled negative control (Panel **B**) was transfected into Neuro-2A cells at the designated concentrations of siRNA using Lipofectamine 3000. Transfections were incubated at 37 °C, 5% CO_2_ for 48 h prior to trypan blue exclusion assay using the Bio-Rad TC20 automated cell counter. Data are shown as mean ± SEM for triplicate wells of triplicate experiments. UT: untransfected. (Panel **C**): KLF15 siRNA reduces HSV-1 productive infection. Neuro-2A cells were grown in MEM containing 2% stripped FBS and transfected with increasing concentrations of KLF15 siRNA duplexes using Lipofectamine 3000 according to manufacturer instructions at 37 °C, 5% CO_2_ for 24 h. Cells were then infected with HSV-1 at an MOI of 1 for 1 h at 37 °C, 5% CO_2_ with rocking. Media was replaced and infected cells incubated for 24 h. The virus in the designated cultures was measured by plaque assays. Data are shown as mean ± SEM for duplicate wells of triplicate experiments. N.P.D.: No plaques were detected; ** *p* < 0.005; *** *p* < 0.001 by student’s *t*-test.

Previous studies demonstrated that cotransfecting GR and KLF15 with BoHV-1 genomic DNA stimulated productive infection more efficiently than KLF15 or GR alone [25]. However, the effects of KLF15 on HSV-1-productive infection had not been examined. Consequently, we examined the effect of silencing KLF15 in productively infected Neuro-2A cells because they are neuronal cells that can be differentiated into dopaminergic-like neurons [34]. Neuro-2A cells can also be readily transfected, whereas human neuronal cells are not readily transfected. Initial studies tested whether the KLF15 siRNA and negative control siRNA influenced cell toxicity. The KLF15 siRNA did not significantly increase cell toxicity of Neuro-2A cells at concentrations up to 50 nm (Figure 1A). Furthermore, control siRNA did not significantly reduce cell viability using 0.1, 10, 25, or 50 nm concentrations (Figure 1B). Western blot analysis demonstrated that the KLF15 siRNA reduced steady-state levels via a dose-dependent response.

Subsequent studies analyzed the effect of HSV-1 infection when the KLF15 siRNA was transfected into Neuro-2A cells and then cells infected with HSV-1 24 h later. At 24 h after infection, the amount of virus in cultured cells was quantified by plaque assays. These studies revealed that when Neuro-2A cells were transfected with 10 and 25 nm KLF15 siRNA, there was a significant reduction in HSV-1 virus production (Figure 1C). When cultures were transfected with 50 nm of the KLF15 siRNA and then infected with HSV-1, there were no detectable plaques at 24 h after infection. In summary, these studies suggested KLF15 stimulated HSV-1 replication in Neuro-2A cells.

### 3.2. KLF15 Steady-State Protein Levels Increase Following HSV-1 Infection

**Figure 2:**
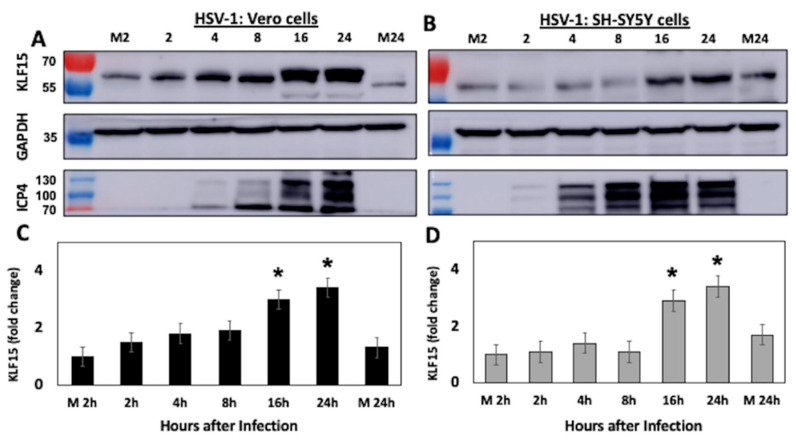
Western blot analysis of KLF15 protein levels during HSV-1 productive infection. Vero (Panel **A**) or human neuroblastoma (SH-SY5Y) cells (Panel **B**) were mock-infected or infected with HSV-1 (MOI = 1 PFU/cell). At the designated times after infection (hours), cells were collected and lysed with RIPA buffer. Proteins were separated by SDS-PAGE, and Western blot analysis performed using the KLF15 polyclonal antibody (1 µg/mL). As a loading control, GAPDH levels were examined. For each lane, 50 µg protein was loaded. Lanes M2 or M24 are 2 or 24 h after mock infection. A representative blot of three independent experiments is shown. The size of molecular weight markers is shown on the left of the blot. (Panels **C**,**D**) show the quantification of KLF15 by densitometry (*n* = 3) using ImageJ 1.26t software, and error bars denote the standard error of the mean (SEM). Significant differences (*p* < 0.05) between cells versus M2 or M24 (students *t*-test) are denoted by an asterisk (*p* < 0.05).

To better understand the relationship between KLF15 and HSV-1 productive infection, KLF15 steady-state protein levels were examined following infection of permissive cells with HSV-1. Vero (Figure 2A) or human neuroblastoma (SH-SY5Y) cells (Figure 2B) were infected with HSV-1 and whole cell lysate, collected at designated times after infection. Western blot analysis indicated KLF15 steady-state protein levels increased as a function of time after infection of Vero cells (Figure 2A). For example, there was an approximately 2-fold increase by 8 h after infection. Furthermore, there was at least a 3-fold increase at 16 and 24 h after infection of Vero cells, which was significantly different relative to Vero cells mock-infected for 2 or 24 h (Figure 2C). At 16 h and 24 h after infection of SH-SY5Y cells, there was also a significant increase in KLF15 steady-state protein levels when compared to cells mock-infected for 2 or 24 h (Figure 2B,D). As expected, ICP4 expression was readily detected by 4 h after infection and increased as viral replication increased. In contrast to KLF15, KLF4 protein levels were not increased following infection of Vero or SH-SY5Y.

**Figure 3:**
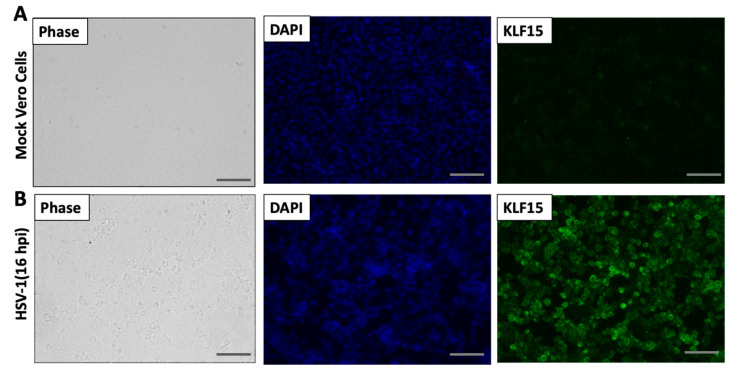
Immunofluorescence studies demonstrated KLF15 protein levels are increased during HSV-1 productive infection. Vero cells were seeded in chamber slides for 24 h and then mock-infected (Panel **A**) or infected (Panel **B**) with HSV-1 (MOI of 1) for 16 h. After washing with PBS three times, cells were fixed with 4% paraformaldehyde in PBS and KLF15 detected by IFA using the KLF15 specific antibody (green). DAPI staining was used to detect nuclear DNA (blue). Images were obtained by performing confocal microscopy (Leica). These images are representative of three independent experiments. (Scale bars are 100 µm).

Immunofluorescence was subsequently performed to compare KLF15 protein expression in infected Vero cells at 16 h after infection versus mock-infected cells (Figure 3). Relative to mock-infected cells (Figure 3A), KLF15 protein expression was readily detected at 16 h of infection (Figure 3B). Collectively, these studies indicated HSV-1-productive infection increased KLF15 steady-state protein levels at late times after infection of Vero and SH-SY5Y cells.

### 3.3. KLF15 Steady-State Protein Levels Increase Following BoHV-1 Infection

**Figure 4:**
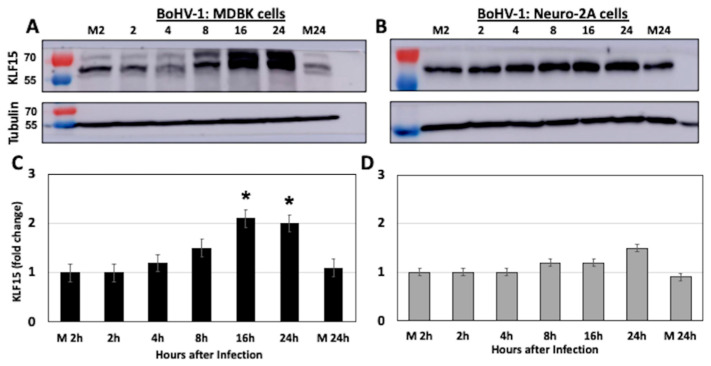
Western blot analysis of KLF15 protein levels during BoHV-1 productive infection. MDBK cells (Panel **A**) or Neuro-2A cells (Panel **B**) were mock-infected or infected with BoHV-1 (MOI = 1 PFU/cell). At the designated times after infection (hours), cells were collected, proteins separated by SDS-PAGE, and Western blot analysis performed using the KLF15 polyclonal antibody (1 µg/mL). As a loading control, tubulin levels were examined. For each lane, 50 µg protein was loaded. Lanes M2 or M24 are 2 or 24 h, respectively, after mock infection. The position of molecular weight markers is shown to the left of the Western Blot. (Panels **C**,**D**) summarize the quantification of KLF15 by densitometry (*n* = 3) using ImageJ 1.26t software, and error bars denote the standard error of the mean (SEM). Significant differences (*p* < 0.05) between cells versus M2 or M24 (students *t*-test) are denoted by an asterisk (*p* < 0.05).

**Figure 5:**
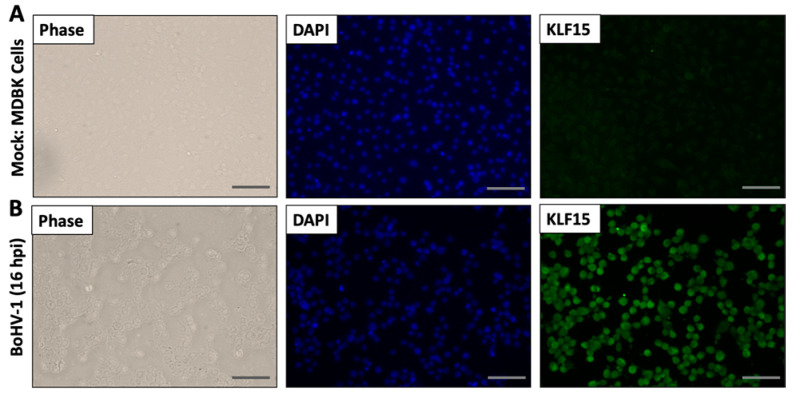
Immunofluorescence studies confirmed KLF15 protein levels increased during BoHV-1-productive infection. MDBK cells were seeded in chamber slides for 24 h and then mock-infected (Panel **A**) or infected (Panel **B**) with BoHV-1 at an MOI of 1 for 16 h. After washing with PBS three times, cells were fixed with 4% paraformaldehyde in PBS and KLF15 detected by IFA using the KLF15 antibody (green). DAPI staining was used to detect nuclear DNA (blue). Images were obtained by performing confocal microscopy (Leica). These images are representative of three independent experiments. (Scale bar, 100 µm).

Bovine kidney (MDBK) or mouse neuroblastoma (Neuro-2A) cells were infected with BoHV-1 and whole cell lysate, collected at the designated times after infection. Western blot studies revealed significantly higher KLF15 steady-state protein levels at 16 and 24 h after infection (Figure 4A,C). The results in MDBK cells were compared to Neuro-2A cells because BoHV-1 does not replicate at high levels in Neuro-2A cells [35]. In contrast to MDBK cells, KLF15 steady-state protein levels (Figure 4C) were not significantly higher after infection (Figure 4D). When compared to mock-infected cells (Figure 5A), immunostaining confirmed an increase in KLF15 expression in infected MDBK cells (Figure 5B), which was consistent with Western blot studies (Figure 4A). In summary, these studies demonstrated there is a correlation between efficient BoHV-1-productive infection and increased KLF15 protein steady-state levels at late times after infection.

### 3.4. Analysis of KLF15 Localization Following Infection

**Figure 6:**
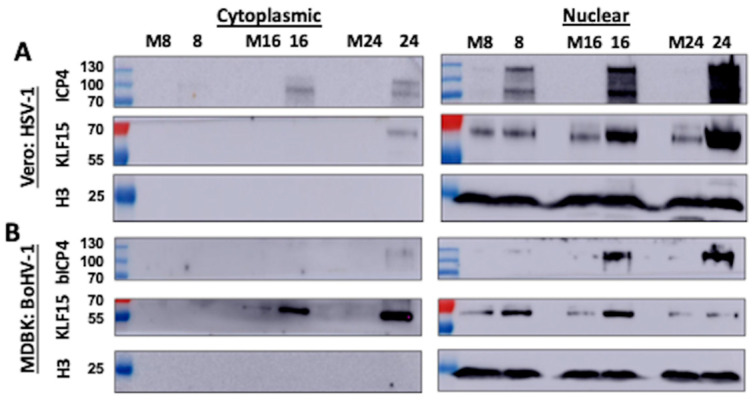
Localization of KLF15 after infection of permissive cells with HSV-1 or BoHV-1. (Panel **A**): Vero cells were mock-infected or infected with HSV-1 at an MOI of 1 for 8, 16, or 24 h. Cells were harvested, and cell fractionation, conducted as described in the materials and methods section. Cytoplasmic and nuclear fractions (50 µg) were analyzed by Western blot using the KLF15 or ICP4 antibody (1 µg/mL). As a control for nuclear proteins, the respective fractions were probed with an antibody directed against Histone H3, which was diluted at 1:500. (Panel **B**): MDBK cells were infected with BoHV-1 at an MOI of 1 for 8, 16, or 24 h. Cellular fractions were analyzed as described in panel A. Lane M is the cell lysate derived from mock-infected cells: numbers denote the time (hours) after mock-infection. A representative blot from two independent studies is shown.

To test whether KLF15 is localized in the nucleus following productive infection with HSV-1 and/or BoHV-1, biochemical fractionation studies were conducted in Vero and MDBK cells at 8, 16, and 24 h after infection. These cell lines were used because they are permissive for the respective viruses. The KLF15 protein was primarily detected in nuclear extracts of Vero cells infected for 8, 16, or 24 h after HSV-1 infection (Figure 6A). Conversely, KLF15 was detected in the nuclear and cytoplasmic extract following BoHV-1 infection at 16 and 24 h (Figure 6B). In fact, KLF15 was primarily detected in the cytoplasmic extract 24 h after infection with BoHV-1. However, KLF15 was primarily detected in the nucleus of MDBK infected cells 8 h after BoHV-1 infection. As expected, ICP4 (Panel A) and bICP4 (panel B) were primarily detected in the nucleus following infection. To ensure biochemical fractionation did not lyse the nuclei of infected cells, histone H3 localization was examined. Histone H3 was detected only in the nucleus after infection with HSV-1 (Figure 6A) or BoHV-1 (Figure 6B), which was expected. In summary, this study revealed KLF15 accumulated in the nucleus of HSV-1 infected cells: conversely, KLF15 primarily localized to the cytoplasm and, to a lesser extent, in the nucleus of MDBK cells at 16 and 24 h after BoHV-1 infection.

### 3.5. KLF15 Promoter Activity Is Stimulated by HSV-1 Infection and ICP0

**Figure 7:**
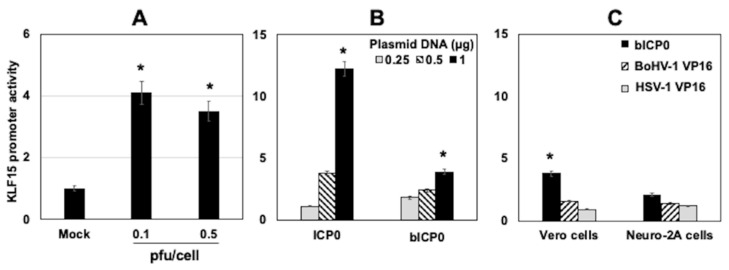
KLF15 promoter was activated during productive infection and ICP0. (Panel **A**): Vero cells were transfected with 1 µg of the pGL4.20-hKLF15 promoter-luciferase construct using Lipofectamine 3000. Twenty-four hours after transfection, cells were infected with HSV-1 at an MOI of 0.1 or 0.5 PFU/cell. At 48 h after transfection, cells were harvested, and protein lysate was subjected to a dual-luciferase assay for measuring KLF15 promoter activity. Promoter activity from mock-infected cells was set at a value of 1, and the other values were compared to mock-infected cells. (Panel **B**): Vero cells were co-transfected with 1 µg of pGL4.20-hKLF15 and increasing concentrations (0.25, 0.5, or 1 µg) of a plasmid expressing HSV-1 ICP0 or BoHV-1 bICP0 using Lipofectamine 3000. (Panel **C**): Vero cells were co-transfected with 1 µg of pGL4.20-hKLF15 and 1 µg of a plasmid expressing BoHV-1 bICP0, BoHV-1 VP16, or HSV-1 VP16 using Lipofectamine 3000. Basal promoter activity from cells transfected with the KLF15 promoter and empty plasmid was set at a value of 1, and other values were compared to this sample (Panel **B**,**C**). The results are the average of *n* = 4 (Panel **A**) or *n* = 3 (Panel **B**,**C**) independent experiments. Error bars denote the standard error of the mean (SEM). A significant difference (*p* < 0.05) between KLF15 promoter activity relatives (students *t*-test) is denoted by an asterisk.

Additional studies tested whether HSV-1 and certain viral transcriptional regulators stimulated KLF15 promoter activity. Vero cells were initially transfected with a luciferase reporter construct containing the human KLF15 promoter region [32]. Cells were subsequently infected with HSV-1 at an MOI of 0.1 or 0.5 PFU/cell for 24 h. HSV-1-infection-stimulated KLF15 promoter activity was approximately 4-fold in Vero cells, which was significantly higher than in mock-infected cells (Figure 7A). Similar studies were attempted in MDBK cells. However, the transfection efficiency of MDBK cells was very low, which made it difficult to perform this experiment.

The studies described in Figure 7A suggest a viral encoded protein-stimulated KLF15 promoter activity, which correlated with increased KLF15 steady-state protein levels following infection. ICP0 and bICP0 are promiscuous transactivators [36], suggesting these viral proteins may stimulate KLF15 promoter activity. To test this prediction, Vero cells were co-transfected with the KLF15 promoter construct and increasing concentrations of a plasmid that expresses ICP0 or bICP0. The HSV-1 ICP0 expression plasmid transactivated the KLF15 promoter more than 11-fold in transfected Vero cells (Figure 7B). Although the BoHV-1 bICP0 expression plasmid transactivated the KLF15 promoter approximately 4-fold, it was not as efficient as ICP0. As a comparison to ICP0 and bICP0, the effect of VP16 on KLF15 promoter activity was also examined. In contrast to ICP0 and bICP0, VP16 encoded by HSV-1 or BoHV-1 did not significantly transactivate the KLF15 promoter in Vero or Neuro-2A cells (Figure 7C). Collectively, these studies demonstrated ICP0 and, to a lesser extent, bICP0 transactivated the KLF15 promoter in the absence of other viral genes.

## 4. Discussion

KLF family members, including KLF15, belong to the Sp1 transcription factor family [37,38]. In fact, Sp1 directly activates HSV-1 gene expression because there are many Sp1 binding sites in the genome [39]. The BoHV-1 genome also contains many Sp1-binding sites, suggesting Sp1 can transactivate key viral promoters. KLF family members bind GC- or CA-rich motifs, including certain motifs that contain consensus Sp1-binding sites [37,40]. Since BoHV-1 and HSV-1 are GC-rich genomes, KLF15 may stimulate certain viral promoters. GR and KLF15 regulate gene expression dynamics and integrate signals by a feed-forward transcription loop in response to stressful stimuli [41,42,43]. The hallmark of a feed-forward transcription loop is the primary factor (GR, for example) stimulates KLF15 expression [44,45,46,47,48,49]. Consequently, GR and KLF15 activate a novel gene expression program relative to GR or KLF15 [41,42,43]. Interactions between GR and KLF15 are also important for the feed-forward transcription loop to be effective. Since HSV-1 infection is stimulated by corticosteroids [26,27] and can increase GR and NF-kB steady-state protein levels [26], GR and KLF15 may stimulate viral gene expression via a feed-forward transcription loop in certain cell types. GR and KLF15 occupancy of ICP0 promoter sequences [27] occurred prior to ICP4 promoter sequences [50] when cultures are treated with DEX. Furthermore, GR and KLF15 occupy IEtu1 promoter sequences prior to the bICP0 E promoter [51] when cultures are treated with DEX, adding support to the concept these interactions are important. Since silencing KLF15 interfered with HSV-1 replication and over-expressing GR and KLF15 stimulated BoHV-1 replication [25], we predict GR and KLF15 interactions with viral promoters have biological relevance during productive infection and following stressful stimuli.

In the absence of other viral genes, ICP0 and bICP0 transactivated KLF15 promoter activity, which correlated with increased KLF15 steady-state protein levels during productive infection. ICP0 and bICP0 are promiscuous transactivators that apparently do not specifically bind DNA [36,52]. While bICP0 was not as effective as ICP0, it did consistently stimulate KLF15 promoter activity. Conversely, HSV-1- and BoHV-1-encoded VP16 had no effect on KLF15 promoter activity. Based on the known functions of these viral proteins, we suggested two scenarios by which ICP0 and bICP0 stimulated KLF15 promoter activity. First, bICP0 and ICP0 interacted with histone-modifying enzymes, altered histone modification, and promoted histone removal [53,54,55,56], suggesting these viral proteins remodeled chromatin within the KLF15 promoter and, consequently, stimulated promoter activity. Secondly, the ability of bICP0 and ICP0 to function as E3 ubiquitin ligases [52,57,58,59,60,61] might indirectly increase KLF15 promoter activity by degrading proteins that impair KLF15 promoter activity. In the context of productive infection, we cannot rule out the possibility that other viral genes play a role in increasing KLF15 steady-state protein levels.

As pointed out in the introduction, significant differences exist in the organization of the ICP0- and ICP4-coding strategy of BoHV-1 relative to HSV-1. There were several other significant differences that existed between BoHV-1 and HSV-1. For example, the BoHV-1 IEtu1 promoter contained two consensuses GREs that are essential for GR and KLF15 mediated transactivation when cultures are treated with dexamethasone [24,25]. Like the bICP0 E promoter [51], the HSV-1 ICP0 [27] and ICP4 promoters [50] do not contain consensus GREs. However, these promoters were cooperatively transactivated by GR and KLF15, suggesting KLF15, not GR, mediated transactivation of the three promoters that lack consensus GREs. For example, Sp1 sequences and KLF4-binding sites in the ICP4 promoter [50] were critical for GR and KLF15 cooperative transactivation for ICP4 enhancer sequences. Since several KLF15-binding sites have been described [62,63,64,65], merely examining the sequences of given viral promoters might not identify a KLF15 responsive motif.

## 5. Conclusions

The studies in this report demonstrated BoHV-1 and HSV-1 stimulate KLF15 steady-state protein levels during late stages of productive infection. While our studies predicted KLF15 stimulates viral gene expression during productive infection, KLF15 may also enhance additional steps during the late stages of productive infection. Future studies will focus on delineating the mechanism by which KLF15 regulates productive infection as well as certain aspects of reactivation from latency.
